# Percutaneous image-guided ablation of bone and soft tissue tumours: a review of available techniques and protective measures

**DOI:** 10.1007/s13244-014-0332-6

**Published:** 2014-05-17

**Authors:** Dimitrios K. Filippiadis, Sean Tutton, Argyro Mazioti, Alexis Kelekis

**Affiliations:** 1 2nd Department of Radiology, University General Hospital “ATTIKON”, 1 Rimini str, 12462 Athens, Greece; 2 Division of Vascular and Interventional Radiology, Medical College of Wisconsin, 9200 West Wisconsin Av, Milwaukee, WI 53226 USA

**Keywords:** Bone, Soft tissues, Thermal ablation, Cryoablation, Protective measures

## Abstract

**Background:**

Primary or metastatic osseous and soft tissue lesions can be treated by ablation techniques.

**Methods:**

These techniques are classified into chemical ablation (including ethanol or acetic acid injection) and thermal ablation (including laser, radiofrequency, microwave, cryoablation, radiofrequency ionisation and MR-guided HIFU). Ablation can be performed either alone or in combination with surgical or other percutaneous techniques.

**Results:**

In most cases, ablation provides curative treatment for benign lesions and malignant lesions up to 3 cm. Furthermore, it can be a palliative treatment providing pain reduction and local control of the disease, diminishing the tumour burden and mass effect on organs. Ablation may result in bone weakening; therefore, whenever stabilisation is undermined, bone augmentation should follow ablation depending on the lesion size and location.

**Conclusion:**

Thermal ablation of bone and soft tissues demonstrates high success and relatively low complication rates. However, the most common complication is the iatrogenic thermal damage of surrounding sensitive structures. Nervous structures are very sensitive to extremely high and low temperatures with resultant transient or permanent neurological damage. Thermal damage can cause normal bone osteonecrosis in the lesion’s periphery, surrounding muscular atrophy and scarring, and skin burns. Successful thermal ablation requires a sufficient ablation volume and thermal protection of the surrounding vulnerable structures.

**Teaching points:**

• *Percutaneous ablations constitute a safe and efficacious therapy for treatment of osteoid osteoma*.

• *Ablation techniques can treat painful malignant MSK lesions and provide local tumour control*.

• *Thermal ablation of bone and soft tissues demonstrates high success and low complication rates*.

• *Nerves, cartilage and skin are sensitive to extremely high and low temperatures*.

• *Successful thermal ablation occasionally requires thermal protection of the surrounding structures*.

## Introduction

During the last decades, technological evolution in the fields of both imaging and instrumentation has led to the development of minimally invasive percutaneous ablative techniques for the treatment of benign and malignant bone tumours. Nowadays, percutaneous ablation techniques in the musculoskeletal system include chemical ablation (i.e. injection of ethanol, acetic acid), irreversible electroporation (IRE) and thermal ablation [radiofrequency ablation (RFA), microwave ablation (MWA), cryoablation] [1–11]. In the latter techniques, one should also include coblation (radiofrequency ionisation used mainly for tumour decompression), irreversible electroporation (IRE) and MR-guided HIFU (high intensity focus ultrasound, which is totally noninvasive). These techniques may act as first-line therapies in certain pathological entities or as attractive adjuncts to conservative therapy, radiotherapy or surgery in other cases.

Prior to performing any ablation, the interventional radiologist should be aware of the tumour histology (benign or malignant), the patient’s general condition and the degree of bone destruction (will consolidation be needed?) [2]. The final decision for malignant cases is made by multidisciplinary oncologic boards. The aim of the ablation (curative versus palliative) should be precisely defined from the beginning of the strategy planning. According to the standards of practice for bone ablation, indications for curative treatments include benign (osteoid osteoma, osteoblastoma <3 cm in diameter, chondroblastoma) and malignant (slow-growing cancers with <3 lesions of <3 cm in diameter each) lesions [2]. Indications for palliative therapies include pain reduction, tumour debulking, decompression and reduction and/or prevention of impeding pathological fractures (where stabilisation will be necessary in combination with the ablation technique) [1–11].

This article will describe the mechanism of action of different ablative technologies applied to the musculoskeletal system, summarise the data concerning the safety and effectiveness of percutaneous ablation techniques for benign and malignant (primary and metastatic) lesions and describe the necessary protective measures.

## Ablation techniques

### General principles

Ablation in the musculoskeletal system can be quite painful and should be performed under some kind of anaesthesia, ranging from conscious sedation to general anaesthesia. All procedures are performed under extensive local sterility measures and prophylactic antibiotics. Whenever an intact bone cortex is noted the coaxial approach is required; the trocar is either drilled or hammered through the cortex depending on the type of cortical reaction (lytic vs. blastic). Once in position, the needle is removed from the trocar and the ablation instrument is inserted coaxially. It is recommended to pass through the lesion before placing the probe, as the sensitive tip of the ablation probe could be damaged by trying to force it through the lesion. Always remember to move the trocar away from the expected ablation zone in order to avoid having a conductor that will transmit heat from the lesion to the surface, with resultant skin and soft tissues burns. In addition, extreme care should be taken concerning the surrounding structures, especially those sensitive to heat or cold (e.g. nerves). Heating at 45˚C has been shown to be neurotoxic to spinal cord and peripheral nerves [12–14]. Cryoablation can also cause neural damage with temporary neuropraxia occurring at −20˚C and permanent neurological damage at ≤−40˚C [2]. Protective measures include passive thermal protection techniques (thermocouples for temperature monitoring, intraoperative neurological monitoring systems such as neurodiagnostic EEG, EMG and evoked potential electrodes and accessories) or active thermal protection techniques (skin protection, hydrodissection, CO_2_ or air insulation) [2, 15].

#### Ethanol ablation

Access is gained to the lesion through a percutaneously placed needle. Ethanol injection causes cellular dehydration, vascular thrombosis and ischaemia; however, the technique is governed by the disadvantage of unpredictable diffusion [8].

#### Laser ablation

Lasers [neodymium yttrium aluminum garnet (Nd:YAG) diode laser 800–1,000 nm] are coaxially inserted in the tumour and transmit infra-red light energy, which results in protein denaturation and coagulation necrosis [2, 8]. Concerning active protective techniques, gas dissection can be performed with air or CO_2_ (CO_2_ is more soluble and a better insulator than air; it is therefore more commonly preferred), hydrodissection is performed with dextrose 5 % (acts as an insulator as opposed to normal saline, which acts as a conductor). All kinds of skin cooling, thermal and neural monitoring can be performed.

#### Radiofrequency ablation

Straight or expandable percutaneously placed electrodes deliver a high-frequency alternating current, which causes ionic agitation with resultant frictional heat (temperatures of 60–100 ˚C) that produces protein denaturation and coagulation necrosis [8]. Concerning active protective techniques, all kinds of gas dissection can be performed. Hydrodissection is performed with dextrose 5 % (acts as an insulator as opposed to normal saline, which acts as a conductor). All kinds of skin cooling, thermal and neural monitoring can be performed.

#### Microwave ablation

Straight percutaneously placed antennae deliver electromagnetic microwaves (915 or 2,450 MHz) with resultant frictional heat (temperatures of 60–100 ˚C) that produces protein denaturation and coagulation necrosis [8]. Concerning active protective techniques, all kinds of gas dissection can be performed, whilst hydrodissection is usually avoided (MWA is based on agitation of water molecules for energy transmission). All kinds of skin cooling, thermal and neural monitoring can be performed.

#### Cryoablation

Straight percutaneously placed cryoprobes deliver room temperature argon gas (for cooling) and helium gas (for thawing) with two cycles of 10-min freezing separated by a 5-min cycle of active thawing usually constituting a typical ablation session [8]. An advantage of cryoablation is that the ice ball is visible under imaging guidance. Also, the technique is governed by significantly lower peri- and post-procedural pain [5]. Disadvantages include the increased cost and time duration [6]. Concerning active protective measures, all kinds of gas dissection can be performed; however, hydrodissection is contraindicated since fluid will freeze when in contact with the ice ball. All kinds of skin warming, thermal and neural monitoring can be performed.

#### MR-guided HIFU

Focussed ultrasound energy is delivered within the lesion under MR guidace with resultant focal elevated temperatures [8]. An advantage of the technique is the real-time thermal monitoring provided by the MR guidance. MR-guided HIFU sessions are performed with a focussed ultrasound phased-array system for treatment, which is integrated with the MR system. Patients are placed in the optimal position to align the lesion to be treated with the ultrasound transducer located in the MR table. The transducer is housed in an oil bath located in the MR table; a moistened gel pad is used to couple the transducer to the patient’s skin and to eliminate air across the path of the ultrasound beam. Treatment planning is three-dimensional, including a combination of coronal, sagittal and axial sequences with and without fat suppression. In addition, treatment planning takes account of the anticipated energy delivered through the skin to the target lesion. Low subtherapeutic sonications can be used to verify the correct target area and then treatment starts at full therapeutic power.

#### Irreversible Electroporation (IRE)

Each cell membrane point has a local transmembrane voltage that determines a dynamic phenomenon called electroporation (reversible or irreversible) [16]. Electroporation is manifested by specific transmembrane voltage thresholds related to a given pulse duration and shape. Thus, a threshold for an electronic field magnitude is defined and only cells with higher electric field magnitudes than this threshold are electroporated. IRE produces persistent nano-sized membrane pores compromising the viability of cells [16]. On the other hand, collagen and other supporting structures remain unaffected. The IRE generator produces direct current (25–45 A) electric pulses of high voltage (1,500–3,000 V).

### Benign tumours

Osteoid osteoma is a relatively common benign tumour (2-3 % of all bone tumours, 10 % of benign bone tumours) usually seen in children and young adults [17]. Although these small tumours (usually of <1.5 cm diameter) have no or a minimum growth rate, they seem larger in imaging studies because of the surrounding oedema and tissue reaction [17]. Due to the extreme appearance of the surrounding tissues, MRI usually leads to overestimation and occasionally is misleading for the diagnosis; CT seems to be the method of choice for the diagnosis of osteoid osteoma [18]. This benign tumour is quite painful and patients complain of localised pain that is worse in the night and characteristically relieved by NSAIDs (nonsteroid antiinflammatory drugs) [6]. In the literature there are few studies with limited patient numbers reporting disappearance of the pain after conservative therapy even if the imaging findings stay the same with no changes [19–21]. Before deciding to try to treat an osteoid osteoma conservatively, the physician should weigh the potential complications, including not only those from long-term use of NSAIDs but also the chance of muscular atrophy and bone deformity in patients aged <5 years [6, 22].

Radiofrequency ablation was first performed in osteoid osteoma [23, 24]. Ever since, for the therapy of this benign tumour, thermal ablation constitutes a first-line therapy. Concerning osteoid osteoma ablation, throughout the literature there are numerous studies, some with smaller and others with higher numbers of patients, but all of them have quite high pain reduction rates (up to 96 %) and low recurrence rates (∼7 % at 2 years) in common [6, 25–31]. Described thermal ablation techniques for the treatment of osteoid osteoma include the use of monopolar or bipolar RF electrodes, plasma-mediated RF electrodes and laser [32–36]. Recent studies on the use of microwaves and MR-guided HIFU report similar success rates and minimal complications [37–39].

**Fig. 1:**
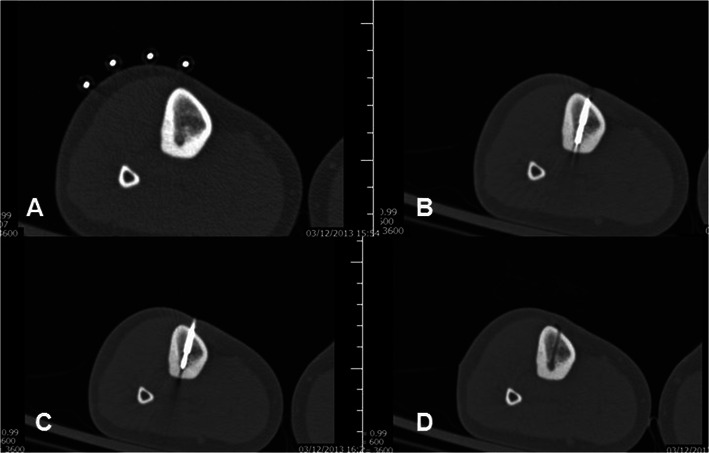
Osteoid osteoma in the tibia: **a** CT axial scan: metallic markers were placed on the patient’s skin. **b** CT axial scan: Once the trocar reaches the lesion, the needle is removed and a bone biopsy needle is inserted coaxially for sampling to verify the diagnosis. **c** CT axial scan: Then, the radiofrequency electrode is inserted into the lesion coaxially and an ablation protocol specific for osteoid osteoma is performed. **d** CT axial scan: Follow-up post trocar removal. Notice the trocar tract, which ends up in the lesion

Percutaneous ablation of osteoid osteoma is performed under CT guidance, extended local sterility measures and antibiotic prophylaxis. Access to the nidus is achieved with a trocar that is either hammered or drilled through the intact bone. Once inside the nidus, a bone biopsy needle can be inserted coaxially and a sample obtained to verify the osteoma diagnosis. Then the electrode is inserted coaxially through the trocar, and ablation is performed with a specific protocol resulting in an ablation zone of ∼1 cm diameter (Fig. 1). Potential complications are rare and include iatrogenic damage to the surrounding nerve root or tissues due to the electrode placement, heat effect and size of the bone necrosis [6]. Follow-up of successful ablation is performed clinically and there is no need for imaging follow-up in asymptomatic patients [6].

Traditional surgical techniques for osteoid osteoma treatment include wide excision removing a bone block, marginal resection of the entire nidus, curettage or high-speed burr techniques [40]. Comparison of these techniques to percutaneous, minimally invasive, imaging-guided ablation favours the latter in terms of minimum trauma, minimum functional restriction and significantly lower cost [40].

**Fig. 2:**
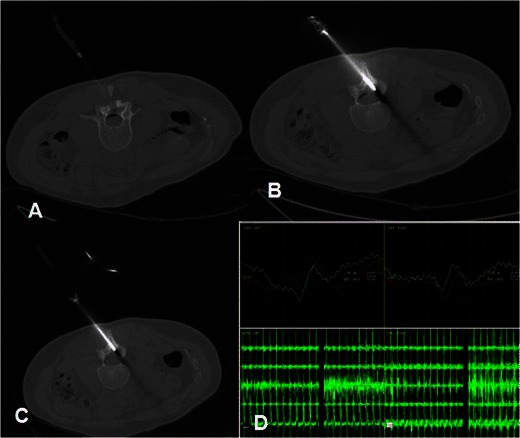
Osteoblastoma in the L2-L3 facet joint: **a** CT axial scan: A 22G spinal needle is inserted inside the epidural space and air is injected through the antimicrobial filter. **b** Axial CT scan: A trocar is drilled till the lesion. **c** Axial CT scan: Coaxially, a bipolar radiofrequency electrode is introduced inside the lesion and the ablation protocol is performed. **d** During the ablation protocol, evoked potentials are used for nerve monitoring

Thermal ablation can be used as a treatment in a variety of benign tumours including osteoblastoma (<3 cm in diameter) (Fig. 2) and chondroblastoma, whilst throughout the literature there are reports of ablation in cases of chondromyxoid fibroma, intracortical chondroma, aneurysmal bone cyst, eosinophilic granuloma and cystic hydroma [2, 6, 41–43]. State-of-the-art reviews report that “essentially any small well defined lesion at imaging can be treated with RF ablation” [6].

Recent studies upon cryoablation report promising preliminary results whenever the technique is applied as the treatment in extrabdominal desmoids tumours [44, 45]. This kind of treatment for soft tissue tumours seems to be governed by high efficacy concerning local tumour control and pain reduction and at the same time by the reduced complication rate and convalescence rate post-therapeutically [44, 45].

### Malignant tumours

The most common malignant bone tumours are metastatic lesions; any malignant neoplasm possesses the capacity to metastasise in the musculoskeletal system [46]. In the general population carcinomas of the breast, prostate, lung and kidney constitute, in decreasing order, ∼75 % of skeletal metastasis cases whilst carcinomas of the prostate, lung and bladder are more common in males and carcinomas of the breast and uterus more common in females [46]. Osseous metastatic disease especially when lytic lesions are involved can result in significant pain and mobility impairment. In most cases the therapeutic goal is palliation with resultant pain reduction and mobility improvement. Alternative therapeutic goals in palliative treatments include tumour reduction/decompression and bone consolidation. Potential therapies include surgery, embolisation, chemotherapy, osteoplasty, ablation, radiotherapy and palliative analgesics. External beam radiotherapy seems to provide at least partial pain relief in 50 % and 80 % of patients with only 30 % reporting complete pain relief [47]. In addition, radiotherapy may result in osteonecrosis or neural damage [48].

Apart from completely eliminating small oligometastatic disease (<3 lesions each measuring <3 cm in diameter), ablation is mainly used for achieving pain reduction to necrotise the interface between the tumour and the pain-sensitive periosteum [2, 3, 5, 6]. Alternative pathophysiologic explanations for pain reduction post ablation include decompression of the tumour volume, a decrease in nerve-stimulating cytokines released by the tumour and inhibition of osteoclast activity [8, 49]. Proper patient selection cannot be emphasised enough; since ablation is a local therapy only patients with localised, at least moderate (>4/10 numeric visual scale units) pain should be treated. Absolute contraindications include coagulopathy disorders, skin infection, immunosuppression and the absence of a safe path to the lesion [50].

**Fig. 3:**
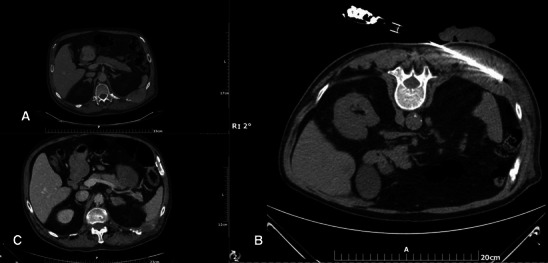
**a** Painful soft tissue mass infiltrating the left T10 posterior rib. **b** A microwave antenna is percutaneously inserted inside the mass. Due to the proximity to the skin a sterile glove filled with cold water is placed over the skin. **c** CT axial scan 3 months after the ablation session: Notice the significant size reduction of the mass along with new bone formation around the left T10 posterior rib (similar to heterotopic ossification)

Percutaneous ablation of malignant metastatic lesions is performed under imaging guidance, extended local sterility measures and antibiotic prophylaxis. Whenever the ablation zone is expected to extend up to 1 cm close to critical structures (e.g. the nerve root, skin, etc.), all the necessary thermal protection techniques should be applied (Fig. 3).

**Fig. 4:**
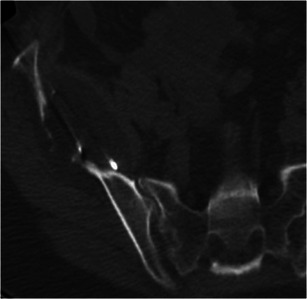
Melanoma metastasis in the right iliac bone: Cryoablation was performed with five cryoprobes inserted; notice how the iceball is visualised on the CT scan as a hypodense structure

Recent reviews and studies on ablation report promising results whenever the technique is applied as treatment in secondary lesions located in the soft tissues resulting in local tumour control and pain reduction with reduced complication rates and convalescence rates post-therapeutically [8, 45, 50–53]. Similarly, studies in the literature on percutaneous ablation (RFA, MWA, MR-guided HIFU and cryoablation) of metastatic painful bony lesions report significant pain reduction with minimal complications (Fig. 4) [11, 51–53]. To date, there seems to be no difference in the efficacy of one technique over the other [9].

A recent application of ablation techniques, more specifically of HIFU, is its use as a limb-salvaging treatment in patients with osteosarcoma with resultant changes in symptoms and survival time [54]. However, further and more extended studies are necessary for confirmation.

The cost of tumour necrosis in bone lesions treated with ablation techniques is bone weakening. In order to avoid post-therapeutic osteonecrosis with pathologic fractures, cement augmentation should be combined with ablation especially in the spine and other weight-bearing areas [2, 3, 5, 6, 8, 9, 50, 55]. Furthermore, in certain locations, due to the direction of the forces applied upon weight bearing, cement augmentation might be insufficient and should be combined with further augmentation with metallic instrumentation; preliminary studies upon such combinations report very promising results [56–58]. Whenever combining ablation with cementation at the same session, it is necessary to allow enough time for the tumour temperature to return back to normal in order to avoid untimely cement polymerisation [50].

In order to enhance local tumour control, ablation can be combined with transarterial chemoembolisation as well (especially for hypervascular lesions usually originating from thyroid, renal cell or hepatocellular carcinomas) [59, 60]. Whenever combining ablation with TACE or any other embolisation technique, the intravascular technique usually precedes the ablation with/without cement augmentation [50].

## Protective techniques

Protective measures include various techniques of insulation and temperature or nerve function monitoring aiming at protecting vulnerable structures. Passive thermal protection techniques include continuous monitoring of the temperature in the area of interest by thermocouples or of nervous function by monitoring systems such as neurodiagnostic EEG, EMG and evoked potential electrodes with accessories [61, 62]. Measurement of the temperature in proximity to a neural structure or of the nerve’s functional ability during the ablation provides valuable information for a safe and efficient session. Heating over 45 °C or cooling below 10 °C can be neurotoxic to the spinal cord and the peripheral nerves [2].

Active thermal protection techniques include gas dissection, hydrodissection and warming/cooling modes for skin protection (subcutaneous fluid injection, application of a sterile glove with warm or cold saline).

During gas dissection, CO_2_ or air is injected by means of a 22G spinal needle for dissecting vulnerable structures away from the ablation zone. CO_2_ is a better insulator than air, which is less soluble and might result in emboli formation [61, 62]. For both gases use of an antimicrobial filter is optional. Dissection by gas is not governed by heating or cooling properties but it is only used in order to increase the distance between the ablation zone and a certain structure [61, 62]. In addition, during the ablation session the injected gas might be absorbed and therefore additional injection might be needed. Gas dissection is the active insulation of choice whenever cryoablation is performed and in most cases of microwave ablation. Gas dissection increases the distance between the expected ablation zone and close nerve structures; in addition, it is more commonly preferred over fluid dissection whenever a spinal or paraspinal lesion is to be ablated and the ablation zone is expected to extend near the epidural space.

Fluid dissection creates the distance necessary for a safe ablation zone, provides cooling or warming (depending on the fluid’s temperature) as well as an occasional insulating effect. In bone and soft tissue ablation this technique can be used in epidural (nerve protection) or articular (cartilage protection) spaces. In addition, it can increase the distance between the expected ablation zone and a close nerve structure. For increased visualisation a small amount of contrast medium can be diluted in the fluid [61, 62]. Whenever fluid dissection is required in ablation performed by radiofrequency energy, dextrose in water (D/W 5 %) is preferred over saline solution because of the latter’s high electrical conductivity [61, 62]. For microwave ablation, any fluid can be used as long as it is distributed at least 2 cm away from the active tip of the antenna. Fluid dissection cannot be used in cryoablation because the used fluid freezes when it comes in contact with the ice ball.

If ablating superficial tumours the skin should be protected in order to avoid and prevent painful burns or frostbite. Skin seems to tolerate low better than high temperatures [61, 62]. Techniques for preventing skin lesions during ablation include positioning sterile gloves containing warm or cooled fluid over the expected ablation zone (for cryoablation and RFA/MWA respectively). In addition, subdermal injection of local anaesthetic or D/W 5 % increases the distance between the ablation zone and skin.

## Conclusion

Percutaneous ablation techniques constitute a safe and efficacious minimally invasive therapy for the treatment of osteoid osteoma and an attractive adjunct in the therapeutic armamentarium of small benign lesions or of oligometastatic bone disease. In addition, thermal ablation can act as palliative therapy in the rest of the cases. Primary or metastatic malignant bone lesions (especially osteolytic) can become very painful. The therapeutic armamentarium includes conservative therapy (analgesics), surgery, chemotherapy, radiotherapy, embolisation, ablation and cementation. External beam radiotherapy, although used frequently, provides moderate pain relief. Tumour necrosis by means of percutaneous ablation techniques seems an effective, safe and feasible technique for the treatment of painful bony lesions. Ablation (RFA, MWA, cryoablation) can treat such lesions and provide local tumour control, performed either alone or in combination with surgical resection or other percutaneous techniques. Specifically for the spine and other weight-bearing locations, ablation should be combined with cement augmentation and occasionally also with further augmentation with metallic instrumentation (which can also be percutaneous). Protection of surrounding sensitive or critical structures is necessary whenever the ablation zone is expected to extend close to these structures. Thorough knowledge of each ablation technique and of all available protective techniques (both passive and active, and in addition how they can be combined) is mandatory for a safe and successful procedure.
